# A Bayesian Geostatistical Moran Curve Model for Estimating Net Changes of Tsetse Populations in Zambia

**DOI:** 10.1371/journal.pone.0096002

**Published:** 2014-04-22

**Authors:** Luigi Sedda, Cornelius Mweempwa, Els Ducheyne, Claudia De Pus, Guy Hendrickx, David J. Rogers

**Affiliations:** 1 Department of Zoology, University of Oxford, Oxford, United Kingdom; 2 Department of Veterinary and Livestock Development, Ministry of Agriculture and Co-operatives, Lusaka, Zambia; 3 Avia-GIS, Zoersel, Belgium; 4 Animal Health Department, Institute of Tropical Medicine, Antwerp, Belgium; University of Athens, Medical School, Greece

## Abstract

For the first time a Bayesian geostatistical version of the Moran Curve, a logarithmic form of the Ricker stock recruitment curve, is proposed that is able to give an estimate of net change in population demographic rates considering components such as fertility and density dependent and density independent mortalities. The method is applied to spatio-temporally referenced count data of tsetse flies obtained from fly-rounds. The model is a linear regression with three components: population rate of change estimated from the Moran curve, an explicit spatio-temporal covariance, and the observation error optimised within a Bayesian framework. The model was applied to the three main climate seasons of Zambia (rainy – January to April, cold-dry – May to August, and hot-dry – September to December) taking into account land surface temperature and (seasonally changing) cattle distribution. The model shows a maximum positive net change during the hot-dry season and a minimum between the rainy and cold-dry seasons. Density independent losses are correlated positively with day-time land surface temperature and negatively with night-time land surface temperature and cattle distribution. The inclusion of density dependent mortality increases considerably the goodness of fit of the model. Cross validation with an independent dataset taken from the same area resulted in a very accurate estimate of tsetse catches. In general, the overall framework provides an important tool for vector control and eradication by identifying vector population concentrations and local vector demographic rates. It can also be applied to the case of sustainable harvesting of natural populations.

## Introduction

There are several ways of analysing animal and plant population data. The simplest involves an actuarial approach as applied to human populations where the likelihood of death and the expectation of future life are assumed to depend only upon age. For many animal and plant populations, however, mortality also comes about from both density independent and density dependent effects and the simple actuarial approach is no longer valid. [1] developed an alternative life-table approach in which the contributions of both density dependent and independent mortalities to changes in total generation mortality were examined over several generations; they defined the ‘key factor’ as the mortality mostly responsible for changes in the total generation mortality over time. A third approach to analysing population data is due to the fisheries research of [2] who produced stock recruitment curves by plotting successive (usually annual) fish counts against each other on a graph (the present year/generation on the x-axis and the next year/generation on the y-axis). The simplest sort of Ricker curve rises rapidly from the origin (when stock numbers increase rapidly from low densities) but eventually turns over as density dependent population limitation becomes important; in an ideal world fishing removes those individuals that would otherwise die through density dependent losses, without affecting the standing crop. Whilst Varley and Gradwell’s life-table approach [1] was developed for species with non-overlapping generations, the Ricker curve [2] can also be applied to species with overlapping generations. Rogers, studying tsetse flies in Africa [3], was the first to connect these two approaches by applying the density dependent and density independent losses concepts of the Varley and Gradwell approach to the logarithmic form of Ricker curve, called a Moran curve (logarithms were necessary because Varley and Gradwell’s mortalities were expressed logarithmically, as ‘k-values’). Tsetse have continuous overlapping generations and the Moran curve approach was shown, through its application to the output of a simple tsetse population model, to be able to quantify (as k-values) the mean monthly density dependent and density independent losses of tsetse in Nigeria and Zambia.

For any population to persist or increase locally total annual fertility must equal or exceed total annual density independent mortality; at equilibrium, the balance of production (fertility minus density independent mortality) is then accounted for by density dependent mortalities (due to competitors, predators or parasites). If inescapable density independent losses exceed fertility, the population will be in continuous decline until it becomes extinct. Such populations can persist in an area only if they are periodically ‘topped up’ from elsewhere, by immigration.

Application of the Moran curve approach to the analysis of field data involves making some assumptions about population fertility which, for simplicity, is usually assumed constant. In reality, tsetse fertility depends mostly upon temperature [4], so the assumption of constancy in Moran curve analysis means that variations in fertility are treated as variations in mortality; this is unlikely to be a serious problem because natural variations in fertility are very much less than natural variations in mortality. The net change in the population’s demographic rates (hereafter simply “net change”) is the difference between fertility and population losses (sum of the density dependent and density independent mortality).

Moran curve analysis (see Appendix S1 for a complete description of the method and its application to tsetse fly sample data) provoked criticism on several fronts, mainly targeted at the subjectivity in choosing the intercept on the y-axis of the Moran plot (determined by the fertility rate) and the density at which density dependent effects begin to become important – the turnover point in the standard Ricker curve [5]. In this paper, we aim: (i) to develop a less subjective Moran curve analysis; (ii) to quantify the tsetse population net change; (iii) to estimate the strength of density dependent effects, the importance of which for tsetse has previously been questioned [6]; and (iv) to demonstrate the advantages for control techniques in identifying both the times (seasons) and places in which to concentrate tsetse suppression activities.

There are other options to analyse population models, for example the Bayesian state-space “normal dynamic linear models” proposed by [7] and applied to the Ricker model. This advanced approach allows the inclusion in the model of observational error (related to the sample data) and process error (related to the functions applied in the model), and the extension of time series analysis to nonstationary and non linear models [8]–[11]. Bayesian state-space models are at present one of the best options for population models. Our aim here, however, is to consider a model with explicit spatio-temporal covariance and population parameters estimated from the Moran curve.

## Methods

### Ethics Statement

The sampling at each location was authorized by the Department of Veterinary and Livestock Development, Zambia. The field studies did not involve endangered or protected species but only tsetse flies. This is not a vertebrate study.

### Field Methods

Analysis was applied to data for tsetse flies (*Glossina morsitans morsitans* Westwood) from South-Eastern Zambia (field data provided in Appendix S6). The sampling scheme was both spatial and longitudinal, and involved man-baited black screens carried on fixed fly-rounds [12], which are commonly used for sampling this species of tsetse (see Appendix S2 for a full description of the sampling method and the geographic area analysed). Sampling was carried out in four sites (listed from North to South): Lusandwa, Kasamanda, Zinaka and Chisulo (Table 1 and Appendix S2). In each site there were two sampling transects arranged in opposite directions and with stopping points at 100 m intervals. *G. morsitans* are attracted to moving black screens and follow the catching party without necessarily landing on the humans or the screen. Periodic stops are therefore used to sample with small fly nets both the following flies and flies landing on either the screen or the surrounding vegetation.

**Table 1:** Details of the sample sites and data collection periods (from – to).

Site information			Fly-round sampling		Tsetse catches		
Name	Geographic centre	Length	Date	Stops	Males	Females	Total
	(degrees)	(m)	(month/year)				
Lusandwa	31.81(long);-13.70(lat)	6,400	12/06–11/07	64	1,310	523	1,833
Kasamanda	31.90(long);-13.78(lat)	4,200	12/06–11/07	94	9	0	9
Zinaka	31.78(long);-13.86(lat)	8,000	07/06–06/07	116	855	249	1,104
Chisulo	31.86(long);-13.96(lat)	4,000	12/06–11/07	74	218	34	252

‘Stops’ are the number of fly catching points on each fly-round.

Sampling was repeated on average seven times each month. For the present analysis, the monthly catches for each stop were used without distinguishing the sexes, because some catches were very small.

Although the data from Kasamanda contain only few non-zero tsetse catches, this site had a similar number of space and time sampling points (Table 1, column “Stops”) as the other three sites and hence it contributes equally in determining the spatio-temporal correlation.

It is common in analysis of tsetse populations to model only the average catches at each site. Here, in a full spatio-temporal framework, the dataset is not averaged, either in space or time. This enables us to retain all of the population variability within sampling sites. Finally, the same number of visits was used at each stop per month, so the data did not need to be standardised.

### Environmental Correlates

The variables used to interpret the level and variation of the density independent mortality are the day-time land surface temperature (DLST), night-time land surface temperature (NLST) and monthly cattle density (see Appendix S3 for further details of these variables).

### Statistical Method

The Bayesian Geostatistical model applied for estimating tsetse population net changes is a modification of the Moran curve method. Starting from the Moran curve model equation, which is assumed to be applied to each of the stopping points on each fly round:
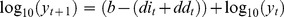
(1)where *b* is the monthly logarithmic fertility rate; *y_t_* and *y_t_*_+1_ are the numbers of flies caught at *n* locations in successive months *t* and *t+1*; *di* is the density independent mortality and *dd* is the density dependent mortality acting at the *S* locations at time *t*, and usually different from one fly round stop to another. The first term on the right hand side 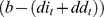 is the net change (difference between fertility rate and population losses) which is zero when the population is in equilibrium.

The model has been extended to a linear regression form with the addition of a spatio-temporal correlation effect, *Z*, and an error component *ε* (observation error):
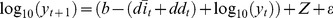
(2)with the following conditionings:



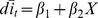





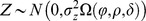





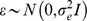
where 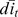 is the density independent mean response from a linear regression of the (matrix of) environmental covariates, *X*, with intercept *β_1_* and regression coefficients *β_2_* (a vector of length equal to the number of environmental covariates). In other words, estimates of *di* are used to fit an environmental linear regression from which *β_1_* and *β_2_* are obtained. The coefficients are then used to back calculate the mean density independent mortality 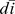. We are interested in the environmental signal of *di*, while its stochasticity will contribute to the other model components (i.e. the spatio-temporal effect and/or the error term).

*Z* is a one column vector with spatio-temporal normally distributed random effects with mean zero and a covariance matrix given by the product of the spatial variance, 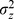, and the correlation matrix, *Ω*; the latter is expressed as a function of the spatial correlation parameter, *φ*, the temporal correlation parameter, *ρ*, and the interaction term, *δ,* as appearing in the double exponential non-separable spatio-temporal function [13] chosen for this analysis:
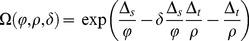
(3)where Δ is the Euclidean distance in space, *s*, or time, *t*, as indicated by the subscript. Finally, ε is the independent and identically normally distributed error.


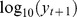
 is Gaussian distributed. Fixing 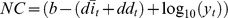 and following the re-parameterisation of the variance parameter of Yan et al. (2007), the model can be re-written as:
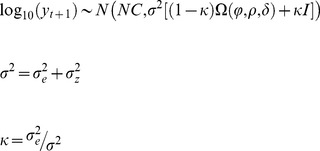
(4)


Hence, the likelihood is:
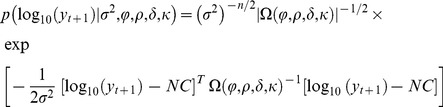
(5)


The posterior density of the model’s unknown parameters, given the observed data *y*, is defined as:
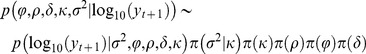
(6)where *π*(⋅) denotes the prior probability of its argument.

In the model the parameters associated with net change are treated differently from those associated with the variance. *di* and *dd* are computed from the fertility rate, *b*, the population size at which the density dependent mortality starts, *a*, and the intensity of density dependent losses expressed as the slope, *α.* Hence, given a set of *b*, *a* and *α*, *di* and *dd* are estimated from the Moran curve model:
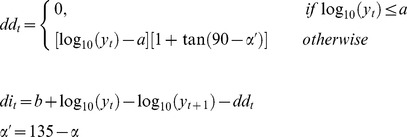
(7)


The losses due to density dependence, *dd*, operate in addition to any density independent losses only above a threshold population size of *a* (the derivation of equation (7) in shown in Appendix S5). Thus the analysis assumes density dependent and independent effects are mutually exclusive and separable (i.e. additive in their effects). This is likely to be an over-simplification; in reality both density dependent and independent mortalities are operating simultaneously. Therefore, as with fertility and mortality, it is likely that there is in reality some mixing of the density dependent and independent mortalities estimated in the way outlined above; our approach will identify only the equivalent density dependent and independent mortalities required additively to bring about the population change actually recorded from one month to the next.

We have produced 27,000 net change candidates from the combinations of values (30) of the parameters:

*b* = sequence of values from 0.2 to 0.4 at intervals of 0.0069.

*a* = sequence of values from 0 to 1.5 at intervals of 0.051.

*α* = sequence of values from 0 to 90 at intervals of 3.1.

For the second set of parameters *φ*, *ρ*, *δ*, *κ*, *σ*,*^2^* we assumed independent priors. The two variances 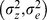 are both Inverse Gamma distributed (this distribution is the conjugate prior for the variance of a Gaussian distribution) of the form IG(*θ*,*γ*) with *θ* = 5 and *γ* = 1 for 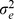 and *θ* = 0.9 and *γ* = 0.25 for 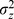. Uniform priors over valid positive limits are defined for the spatio-temporal parameters: spatial range (km), *φ*∼Uniform(0,3); temporal range (months), *ρ*∼Uniform(0,6); interactive term *δ*∼Uniform(0,1). These priors are not the best choice for mixed models without spatial effects [14], [15] because they can lead to an improper posterior, but they are a common choice for their properties (and computation results) in Bayesian mapping [16]. In fact, choosing uniform priors for the spatio-temporal function parameters allows the restriction of their estimate to a small number of them, which leads to a faster and better control of the spatio-temporal covariance by avoiding values associated with the absence of autocorrelation. Alternatively, informative priors can be applied (and need to be investigated), such as Jeffreys priors or Maximal Data Information priors. The posterior samples of *φ*, *ρ*, *δ*, *σ^2^* and *κ* given *y* are obtained from the RAMPS algorithm [17] and the convergence of the MCMC chains was tested with the Gelman-Rubin convergence statistic [18]. The MCMC was run until convergence and until the Monte Carlo error for all the parameters was equal or less than 5% [19]. At the end of MCMC sampling, 200 samples for each parameter were extracted with regular thinning from the respective chains.

In practice, for each net change candidate the RAMPS algorithm finds the Bayesian estimates of the spatio-temporal covariance parameters and observational error. Once the MCMC chains have converged, a sample of the converged chains is taken and used to calculate the model DIC. Therefore the lowest DIC within the 27,000 models (27,000 is the number of net change candidates) was selected and the associated parameters were then employed in the mapping predictions.

The algorithm was built using a series of functions described in Appendix S4.

### Mapping

The fertility rate, the regression coefficients for the density independent effect and the average density dependent effect acting at each month in the model with the lowest DIC were employed to predict the net population change on a grid of points within the study area. Simple kriging was then applied to add a spatio-temporal covariance to this net change. The latter is an interpolator, the estimates of which are obtained from weighted linear combination of the data. Weights are obtained from the spatio-temporal covariance function. In simple kriging the mean is assumed to be known.

### Out-Of-Sample Validation

The model was run on two sites (Lusandwa and Zinaka, the sites with a larger number of tsetse catches) in order to make predictions for a third site (Chisulo). The statistics applied were: mean difference between original and predicted logarithmic tsetse counts (ME) and the mean squared difference between original and predicted logarithmic tsetse counts (MSE). For *s = 1,2,…,S and t = 1,2,…,T* points. The formulae are:
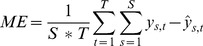
(8)




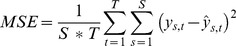
(9)


## Results

The principal aim of this analysis was to estimate and understand the net changes in tsetse populations over space and time. The impact and therefore importance of density dependent mortality can be tested by re-doing the analysis without it, thus considering only density independent mortality. Removing the density dependent component from the model yielded a DIC value of 938, a drastic reduction in model fit when compared with a value of 560 for the full model. This suggests that density dependent losses explain a great deal of the variation of the population data.

The net change in abundance for the study year was slightly greater than zero (0.05), with the largest seasonal value in the hot-dry season (Table 2). Therefore population losses balanced gains and the modelled total population in the area was apparently in equilibrium (increasing by only one individual).

**Table 2:** Mean values of the net change during the three seasons in the four sites in Zambia.

Locations	*Rainy*	*Cold-dry*	*Hot-dry*	*Year mean*
*Lusandwa*	0.080	0.003	0.105	0.061
*Kasamanda*	−0.022	NA	NA	−0.022
*Zinaka*	0.006	0.057	NA	0.032
*Chisulo*	−0.007	0.033	0.107	0.049
*All sites*	0.018	0.028	0.106	0.054

Rainy (January to April), cold-dry (May to August) and hot-dry (September to December) seasons; and the mean values throughout the year.

**Figure 1:**
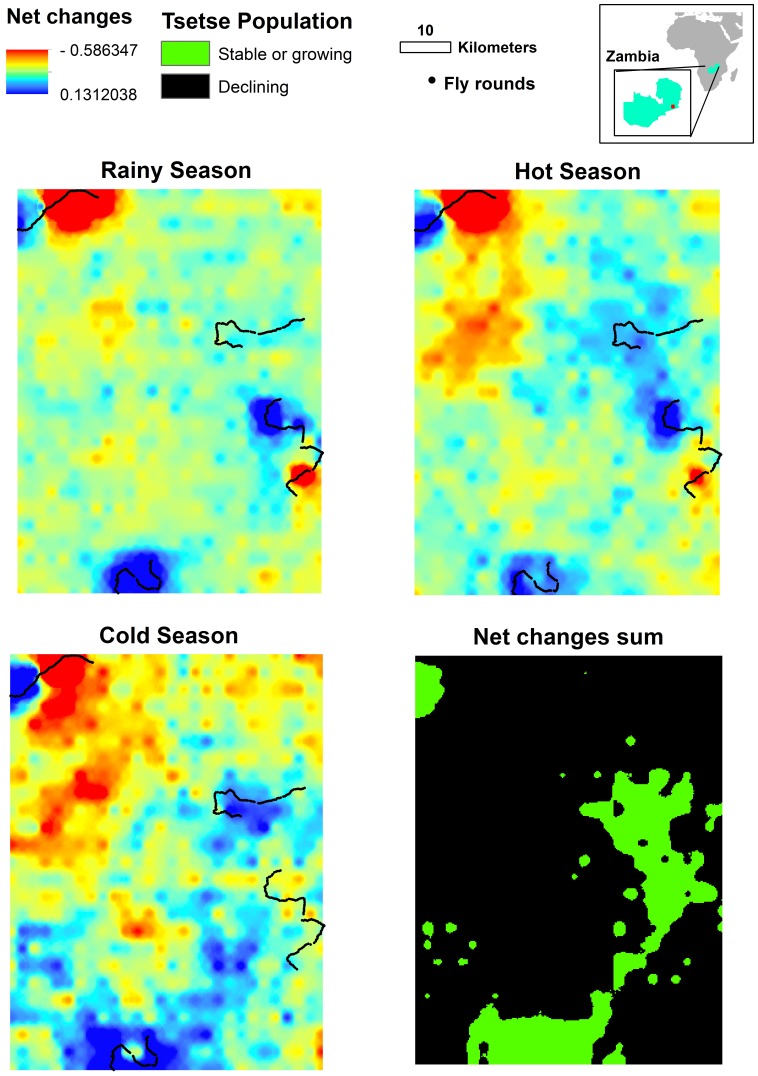
Tsetse population net change in the prediction zone for each season and summed for the entire period. The latter is stable or growing if the net change is equal to or larger than 0; otherwise it is declining. The upper-right corner map shows the position of the predicted area (red square) in Zambia.

The spatio-temporal net change predictions for the three seasons in Zambia are shown in Fig. 1. All three maps show a mixture of high and low net change in both space and time. There is a substantial reduction in net change in the North-West part of the study area between the rainy and cold-dry seasons. The positive large values of net change in the other areas, however, balance these losses and produce an overall positive value of net change for the cold-dry season that is larger than that for the rainy season. The maximum net change occurred in the hot-dry season. The model allows us to identify areas where the tsetse population is always doing well, that is, where the net changes are zero or positive in all three seasons (green areas in the black and green picture of Fig. 1). These areas may be regarded as tsetse ‘sources’ (reservoirs), although a longer sampling period would be needed to determine if this is permanently the case.

The Moran curve parameters associated with the lowest DIC are: 0.23 for fertility rate, 30° for the intensity of density dependence (slope), and 0.46 for the logarithmic population size at which density dependent mortality starts (Table 3).

**Table 3:** Estimates of the different parameters in the model.

Moran curve optimal parameters (4 best models)				
	*1*	*2*	*3*	*4*
*DIC*	560	683	693	701
*b*	0.230	0.401	0.297	0.297
*a*	0.459	0.3380	0.338	0.621
*α*	30	30	30	30
**Coefficients for the density independent linear regression**				
	***Estimate***	***Std. error***	***T value***	***Probability***
*β_1_*	0.642	0.087	7.304	5.19e-13
*β_2_ (LSTD)*	0.011	0.003	3.834	<0.001
*β_2_ (LSTN)*	−0.020	0.005	−4.292	1.92e-05
*β_2_ (CT)*	−0.004	<0.001	−6.531	9.80e-11
**Posterior estimates from the Bayesian components**				
	***Median***	***Mean***	***Std. deviation***	***95% HPD***
*σ* ^2^ *_z_*	0.267	0.283	0.130	(0.184,0.424)
*σ* ^2^ *_e_*	0.060	0.061	0.009	(0.047,0.075)
*φ (km)*	1.568	1.744	1.894	(0.965,3.157)
*ρ (months)*	0.975	0.943	0.082	(0.720,0.999)
*Δ*	0.864	0.836	0.142	(0.467,0.999)

*b*, fertility rate; *a*, population size at which the density dependent mortality effect starts; *α*, angle of the density dependent effect; *β_1_*, intercept of the linear regression for the density independent mortality effect; *β_2_* regression coefficients; *φ*, spatial range; *ρ*, temporal range; *δ*, interactive term for the spatio-temporal effect; 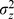, spatial variance; 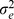, error variance.

The value of 0.23 (the antilog value is 1.69) indicates a maximum rate of population increase in the study area of just under 70% per month, less than the maximum possible value (at slightly higher temperatures) of about 100%, that is, a population doubling per month [3] (female flies produce one full grown larva every 6/7 or more days which pupates more or less immediately and emerges as an adult fly after 20 or more days; all development times are temperature dependent). The slope of the density dependent effect (30 degrees) is characteristic of a population showing under-compensation [20]. This is to be expected of insect species such as tsetse that have very comparatively low fertility rates. Finally, the density dependent mortality starts at a population density of around 2.87 (antilog of 0.46) tsetse flies per stop on the fly rounds. This reflects the usually low values of the catches (90% of the catches were of between zero and three flies per stop; the minimum and maximum catches were, respectively, 0 and 18 tsetse).

The beta coefficients obtained from the regression of density independent losses on climate variables (Table 3) suggest lower mortalities as night-time surface temperatures increased, and when cattle numbers increased, and higher mortalities when day-time land surface temperatures increased. These results are in accordance with the literature on the biology of tsetse, but this analysis quantifies these relationships more clearly than previously.

Finally, the model predicts relatively rapid declines in both spatial and temporal correlations with a spatial range of 1.7 km and a temporal range of 1 month. The average values used to correct the space-time interaction was significantly different from zero, indicating that the two dimensions of space and time cannot be separated, and that the spatial and temporal ranges co-varied (Table 3).

Validation based on applying the net change parameters estimated from Lusandwa and Zinaka to estimate the logarithmic catch data from Chisulo produced accurate statistics: the ME was 0.1 (equivalent to 1.25 tsetse flies) and the MSE 0.35 (equivalent to 2.23 tsetse flies).

## Discussion

**Figure 2:**
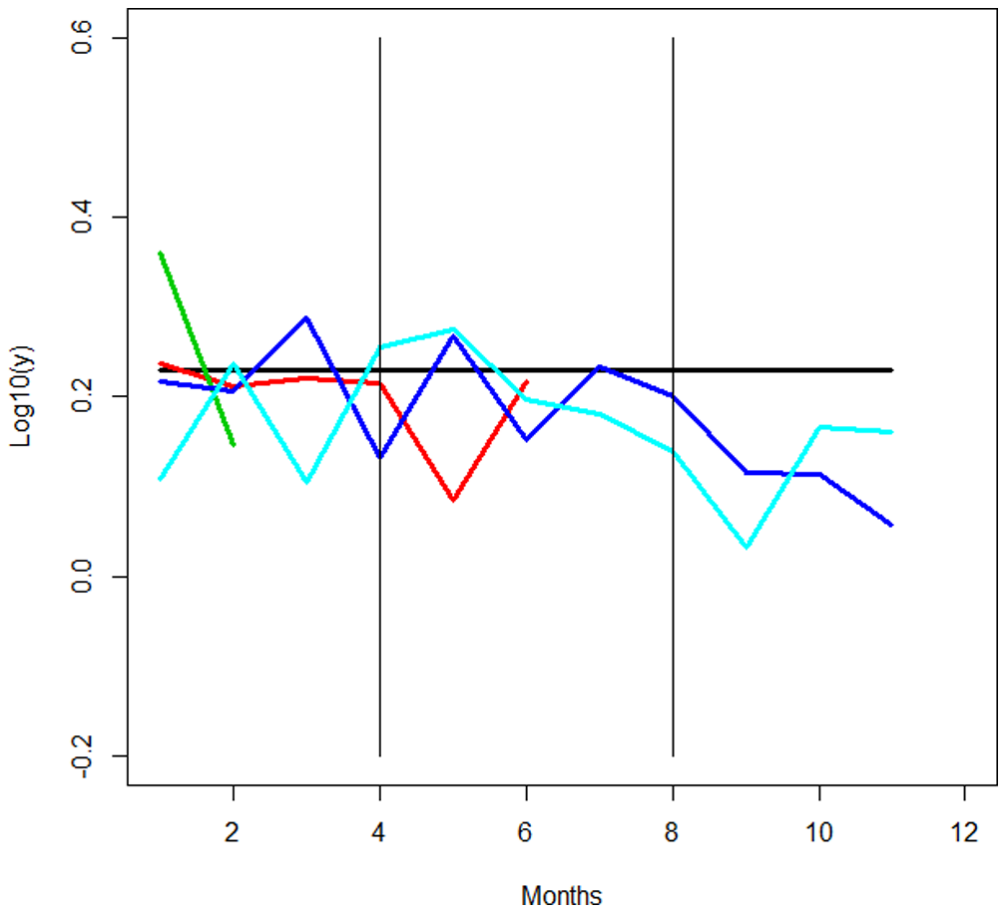
Tsetse population losses (*dd* +*di*) for the different locations: Lusandwa (sky-blue line), Zinaka (red line), Kasamanda (green line) and Chisulo (dark-blue line). The horizontal bold black line at 0.23 is the estimated fertility rate level (assumed constant). The vertical lines delineate the different seasons: rainy (months 1 to 4), cold-dry (months 5 to 8) and hot-dry (months 9 to 11).

The study sites included areas with mean total population losses that were both lower and higher than the estimated fertility rate (Figure 2). Between rainy and cold-dry seasons net changes were negative for most of the sites (except from Zinaka) indicating that the total population was sustained only through immigration; for example in Lusandwa, Chisulo and Kasamanda. In particular the Lusandwa population appears to be maintained only by considerable immigration (from the North-West) occurring mainly in the cold-dry and hot-dry seasons. Such immigration could be from an animal game park near to the northern border of the area [21]. Despite the high immigration rate, the Lusandwa tsetse population is very low, due to low survival rates of both immigrant and resident flies, perhaps because of the limited availability of hosts in Lusandwa. In Zinaka, the partial results (the survey does not cover the whole year) indicates a more stable population with population losses always lower than the fertility rate. Zinaka is a site far from game parks and is unlikely to be affected by immigration. Hence environmental conditions make this area suitable for low density tsetse populations.

Net changes are highest in the hot-dry seasons in Zambia (Table 2). These results are in agreement with previous studies on tsetse populations in Zambia and elsewhere [22], [23], where *G. m. morsitans* is most abundant between the end of the hot dry season and the beginning of the rainy season and less abundant during the cold-dry season because of a combination of climate, vegetation and host grazing patterns.

During the rainy season, density dependent mortality provides an upper limit to fly population density. Density dependent losses from one area may be due not to death but to emigration to other areas which results in immigration (which may also be density dependent) into those areas. Thus density dependent population movement [24], [25] may result in the persistence of tsetse throughout much larger areas. Considerable, periodic large scale movement of tsetse flies has been recorded in the Nguruman area of SW Kenya [26], with possibly the same effects (maintaining populations that otherwise would become seasonally extinct). It appears therefore that in this region of Zambia tsetse seem to show all the characteristics of a metapopulation [27].

It is a matter of some debate whether tsetse populations should be targeted by control when they are most or when they are least numerous; the approach described here can identify these periods in the annual cycle and the additional level of control that needs to be applied to reduce the tsetse population’s rate of increase to the low level required for eventual population extinction.

## Conclusions

The modelling approach suggested here has three key innovations: (i) estimation of the tsetse population’s fertility rate and the levels of density independent and density dependent losses, (ii) implementation of spatio-temporal autocorrelation methods; and (iii) mapping net change, which can be related to environmental or host variables. Even though the optimal search between 27,000 models and the use of Bayesian geostatistics required considerable computer resources and a long processing time, it is an improvement on previous population mapping methods because it quantifies both birth and death rates in a spatio-temporal framework, thus highlighting the levels of additional mortality required, and the times and places to apply them, to reduce the target population to any desired level, and even to extinction.

The present analysis could be improved by adapting the model for simultaneous operation of density independent and density dependent effects. Density dependent losses are likely continuously to be adjusting their impacts as populations rise and fall each month through density independent effects. Obviously, the total losses cannot be less than those actually observed but the allocation of these losses to density dependent or independent causes may change if a continuous approach is adopted.

In conclusion, this research offers a novel approach to estimating key demographic parameters in a geostatistical Bayesian framework. Referring to the origin of the Ricker/Moran curve approach we suggest that this approach could also usefully inform policies for the rational management and harvesting of natural populations such as fish stocks. In the case of tsetse flies, population loss maps are fundamental tools for tsetse control and management. In the case of harvesting, analogous maps can indicate where and when harvesting is most sustainable. Estimating the accuracy and uncertainty of such maps is now possible with widely available, enhanced computational power.

## Supporting Information

Appendix S1 **The Moran curve.**
(DOC)

Appendix S2 **Description of the area investigated and experimental protocol used to sample the tsetse flies.**
(DOC)

Appendix S3 **The environmental variables.**
(DOC)

Appendix S4 **Algorithm components.**
(DOC)

Appendix S5 **Derivation of** **Equation 7** **.**
(DOC)

Appendix S6 **Field data.**
(XLS)
