# Supplementary material for: A Bayesian Geostatistical Moran Curve Model for Estimating Net Changes of Tsetse Populations in Zambia
Source: PLoS One. 2014 Apr 22;9(4):e96002. doi: 10.1371/journal.pone.0096002 (PMC3995969; doi:10.1371/journal.pone.0096002)
Supplement: Appendix S1 — The Moran curve. (DOC) [file pone.0096002.s001.doc]

**Appendix S1: The Moran curve.**

*The use of the Moran curve in tsetse analysis*

Rogers (1979) introduced a new way of calculating tsetse population mortality rates from standard tsetse catch data using Moran curves which are logarithmic forms of Ricker curves that were first developed for fisheries (Ricker 1954). Logarithmic Moran curves lead to estimates of logarithmic mortality rates, or k-values, first applied to insect populations by Varley & Gradwell (1962) and used by them for insect ‘life table analysis’ which is very different from the actuarial life table approach first applied to tsetse by Glasgow (1963), where mortality is solely dependent upon age. In reality for many insect populations, including tsetse, death is not only age related, but also related to biotic and abiotic environmental conditions.

By using the output of a simple tsetse population model, with known input parameters and variables, Rogers showed how the Moran curve approach can be used to estimate seasonal density independent mortality rates of tsetse, and the overall type and level of density dependent mortality operating. Density dependent losses provides an upper limit to population growth. When the population reaches this limit, growth rates fall to replacement values due to a decrease in the birth or immigration rates, or an increase in the death or emigration rates (Hassell 1975). These factors can act individually or in combination, and are always due to various forms of biological agents, such as competition for resources, predation or parasitism. In contrast, density independent mortalities operate entirely independently of density and are often brought about by non-biological agents; for example climatic conditions of a certain severity will kill the same percentage of both high and low tsetse populations. The effect of density independent mortality is to reduce the potential fertility to a lower value (the realised fertility) that takes account of all density independent losses. At equilibrium, density dependent mortality then effectively adjusts itself to balance this realized fertility, so that total fertility equals total (density independent + density dependent) mortality, and the population remains at a constant level (Rogers 1979). It is only the flexibility of density dependent losses that allows populations to persist through time.

Later, Rogers (1990) produced population models based on the Moran curve estimated mortality rates for several species of tsetse in both West and East Africa. These and other studies were able to define a maximum daily tsetse mortality rate, above which populations could not persist (because mortality exceeds fertility); for example, assuming a 30% loss of flies in the puparial stage, the maximum daily adult mortality rate for population persistence is about 0.0355 (Rogers and Randolph 1984). Adult mortality rates continuously in excess of this figure mean that the tsetse population will die out in the area.

*Analytical description of the Moran curve method*


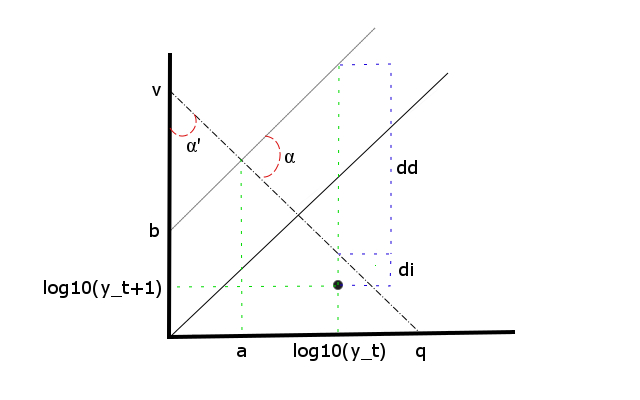


Fig A1. Moran Curve parameters: *y_t* and *y_t+1* are tsetse catches in successive months *t* and *t+1*and are plotted on logarithmic axes.The line from the origin of the axes is the ‘line of equality’ where successive monthly values are the same. The straight line with intercept *b*, parallel to the line of equality, defines the upper limit of population growth from one time interval to the next; line *vq* represents density dependent limitation of population growth; *a* is the point at which the density dependent mortality starts; *di* is one value of density independent mortality; *dd* is the density dependent mortality operating at the same time; and *α* is the angle between *vq* and the line of maximum population growth. The strength of density dependence depends on the value of *α*

The Moran curve has several components (Fig. A1). Its two axes are the numbers of flies caught in successive months (x-axis month *t*; y-axis month *t+1*); both axes are logarithmic. Unlimited population growth at a fixed rate of increase produces on the Moran curve a straight line with a 45 degree-slope and an elevation above the line of equality *w* (where log_10_(y_t_) = log_10_(y_t+1_)) equal to the logarithmic rate of increase (equivalent here to logarithmic birth rate per month, *b*). This maximum reproduction line is shown intercepting the y-axis (x=0) in Fig. A1 at a value of *b*. Density dependent limitation of unlimited population growth causes a break in the maximum reproduction line above a threshold population density of *a* on the x-axis of Fig. A1. Above this threshold density the population trajectory is shown by line *vq* in Fig. A1. Where this line intercepts the line of equality (0,w) determines the equilibrium tsetse population size because, at this point, log10(y_t+1)= log10(y_t). The strength of density dependent mortality is determined by how rapidly the density dependent line diverges from the unlimited population growth line, in other words, by the value of *α* in Fig. A1. The slope of the density dependent linethrough the potential equilibrium point (which is also *α* in Fig. A1) determines the type of local population stability shown around the equilibrium point itself. Relatively low values of *α* (<45 degrees)will give an equilibrium which is under-compensating (i.e. a displacement results in a slow, non-oscillatory return back to equilibrium), whilst higher values give either exact compensation (*α* = 45 degrees and the population returns immediately to its equilibrium after displacement) or increasing degrees of over-compensation (*α* >45 degrees produces oscillations around the equilibrium which can either dampen down or increase without limit, eventually causing population crash and extinction) (Varley et al. 1973).

Use of a straight line for density dependent mortality above *a* is a simplifying assumption which is unlikely to introduce serious errors because tsetse populations show a relatively narrow range of seasonal densities compared with other insects, where non-linear density dependent losses are more likely.

The maximum reproduction line and its density dependent continuation represent population behaviour in the absence of any density independent losses. This latter type of mortality always reduces population growth rates to below those outlined above; i.e. data points usually fall below the maximum values possible. The vertical distance between the actual data point and the theoretical maximum was shown by Rogers (1979) to be a good measure of the density independent mortality operating in any particular month. Since they are calculated on logarithmic axes, these measures of density independent losses are also logarithmic, i.e. they are monthly k-values (Varley et al. 1973). Although they are usually positive (because mortalities always reduce populations), Moran curve analysis occasionally produces negative values for density independent mortality (i.e. when the data point is above the maximum curve defined above), which indicate sampling biases or immigration. On the other hand, very high mortalities may be due to the combined effects of both mortality and emigration, i.e. any source of population loss.

**References**

Glasgow, J.P. 1963. The distribution and abundance of tsetse. Pergamon Press, London.

Hassell, M.P. 1975. Density-dependence in single-species populations. Journal of Animal Ecology 44:283-295.

Ricker, W.E. 1954. Stock and recruitment. Journal of the Fisheries Research Board of Canada 11:559-623.

Rogers, D.J. 1979. Tsetse population dynamics and distribution: anew analytical approach. Journal of Animal Ecology 48:825-849.

Rogers, D.J., and S.E. Randolph. 1984. From a case study to a theoretical basis for tsetse control. Insect Science and its Application 5:419-427.

Rogers, D.J. 1990. A general model for tsetse populations. Insect Science and its Application 11:331-346.

Varley, G.C., and G.R. Gradwell. 1962. The interpretation of insect population changes. Proceedings of the Ceylon Association for the Advancement of Science 18:142-156

Varley, G., G.R. Gradwell, and M.P. Hassell. 1973. Insect population ecology. Blackwell Scientific Publications, Oxford.
