# Supplementary material for: A Bayesian Geostatistical Moran Curve Model for Estimating Net Changes of Tsetse Populations in Zambia
Source: PLoS One. 2014 Apr 22;9(4):e96002. doi: 10.1371/journal.pone.0096002 (PMC3995969; doi:10.1371/journal.pone.0096002)
Supplement: Appendix S2 — Description of the area investigated and experimental protocol used to sample the tsetse flies. (DOC) [file pone.0096002.s002.doc]

**Appendix S2: Description of the area investigated and experimental protocol used to sample the tsetse flies.**


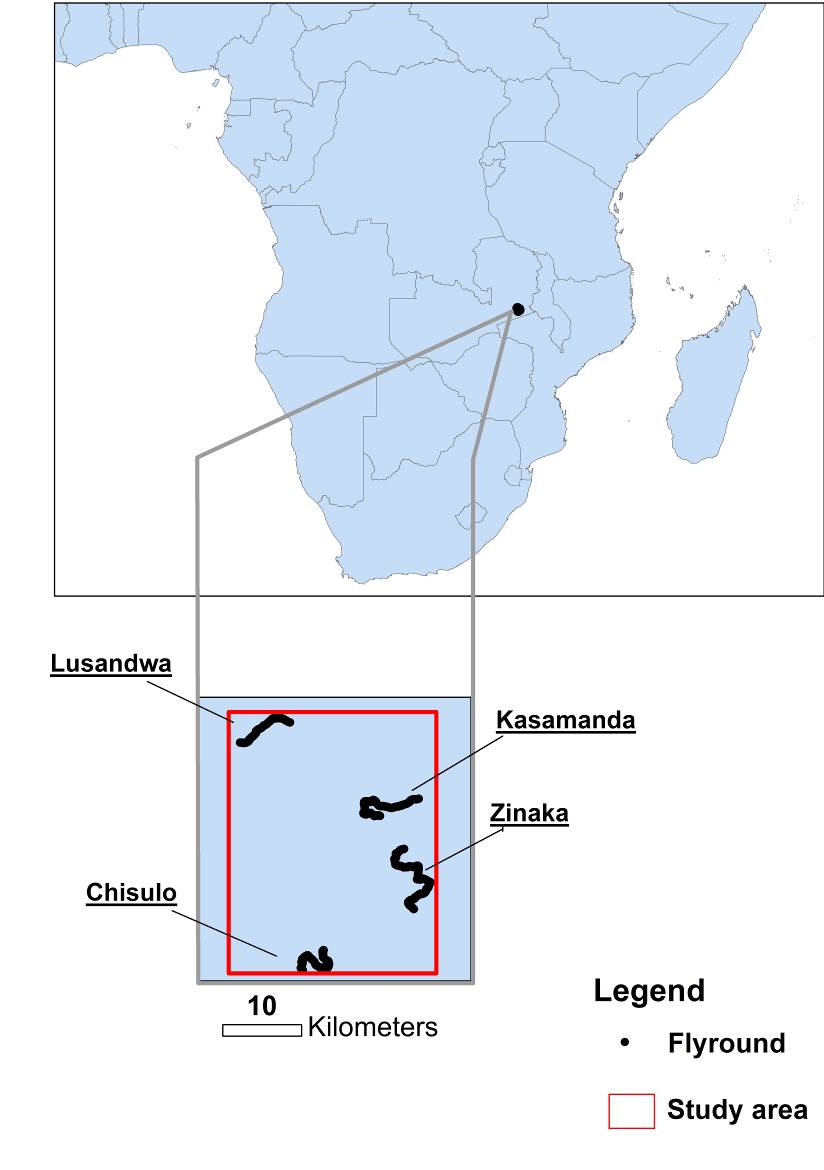


Fig. B1 The study area (red square) and sampling sites (black lines). The area shown is bounded by the following coordinates: lower-left corner, 31.7819 degrees longitude East and -13.9798 degrees latitude South; upper-right corner, 32.0019 longitude East and -13.6858 latitude South.

Tsetse sampling was carried out in an area where land cover changes have resulted in a moderate to severe decrease in natural vegetation and an increase in agricultural activities in the last few decades (Munyati 2000, Yang and Prince 2000, Petit et al. 2001). In this part of Zambia, the commonest tsetse species is *Glossina morsitans morsitans* Westwood (Ford and Katondo 1977), one of the main species responsible for spreading *nagana* and Rhodesian sleeping sickness (trypanosomiasis of domestic animals, especially cattle, and humans, respectively). With two other subspecies, *G. m. morsitans* has a widespread distribution that includes the savannahs of West, Central and East Africa (Bursell 1960, Nash 1969)

The sampling scheme was both spatial and longitudinal, and involved man-baited black screens carried on fixed fly-rounds (Ford et al. 1959, Davies 1967, Van den Bossche and De Deken 2002), which are commonly used for sampling this species of tsetse. Sampling was carried out in the four sites shown in Fig. B1: Lusandwa, Kasamanda, Zinaka and Chisulo. In each site there were two sampling transects arranged in opposite directions and with stopping points at 100 m intervals. *G. morsitans* are attracted to moving black screens and follow the catching party without necessarily landing on the humans or the screen. Periodic stops are therefore used to sample with small fly nets both the following flies and flies landing on either the screen or the surrounding vegetation.

Sampling was repeated on average seven times each month. For the present analysis the monthly catches for each stop (the sum of the tsetse flies caught in a month, defined here and in the tsetse literature as the Apparent Density) were used without distinguishing the sexes, because some catches were very small. The monthly catches at each stop are not averaged or otherwise standardised because we used the same number of visits at each stop per month. If, for example, a stop was sampled 7 times in January but only 6 times in other months, we removed one of the seven samples from that stop’s catches. Averaging or standardisation of the catches would have created some very low numbers (more than half of the dataset records are of zero catches on any single sampling visit), reducing the actual true variance between catches and stops at different times. Moreover, for each stopping point the last zeroes (the zeroes present in the last months of the temporal series) were removed. The presence of zero catches can result in an under-estimation of density independent mortality.

In this area of Zambia there are three seasons in which tsetse mortality might vary: rainy (from January to April), cold-dry (from May to August) and hot-dry (from September to December).

**References**

Bursell, E. 1960. The effect of temperature on the consumption of fat during pupal development in Glossina. Bulletin of Entomological Research 51:583-598.

Davies, H. 1967. Tsetse flies in Northern Nigeria. A handbook for Junior Control Staff, Ibadan University Press.

Ford, J., J.P. Glasgow, D.L. Johns, and J.R. Welch. 1959. Transect fly rounds in field studies of Glossina. Bulletin of Entomological Research 50:275 – 285.

Ford, J., and K.M. Katondo. 1977. Maps of tsetse fly (Glossina) distribution in Africa, 1973, according to sub-generic groups on a scale of 1:5,000,000. Bulletin of Animal Health and Production in Africa 15:187-193.

Munyati, C. 2000. Wetland change detection on the Kafue Flats, Zambia, by classification of a multitemporal remote sensing image dataset. International Journal of Remote Sensing 21:1787-1806.

Nash, T.A.M. 1969. Africa’s bane. The tsetse fly. Collins, London.

Petit, C., T. Scudder, and E. Lambin. 2001. Quantifying processes of land-cover change by remote sensing: resettlement and rapid land-cover changes in south-eastern Zambia. International Journal of Remote Sensing 22:3435-3456.

Yang, J., and S.D. Prince. (2000) Remote sensing of savanna vegetation changes in Eastern Zambia 1972-1989. International Journal of Remote Sensing 21:301-322.

Van den Bossche, P., and R. De Deken. 2002. Seasonal variations in the distribution and abundance of the tsetse fly, Glossina morsitans morsitans in eastern Zambia. Medical and Veterinary Entomology 16:170-176.
