# Supplementary material for: A Bayesian Geostatistical Moran Curve Model for Estimating Net Changes of Tsetse Populations in Zambia
Source: PLoS One. 2014 Apr 22;9(4):e96002. doi: 10.1371/journal.pone.0096002 (PMC3995969; doi:10.1371/journal.pone.0096002)
Supplement: Appendix S3 — The environmental variables. (DOC) [file pone.0096002.s003.doc]

**Appendix S3: The environmental variables.**

Environmental variables used in the analysis included 1km day- and night-time land surface temperature (DLST and NLST respectively) obtained from the MODIS sensor of NASA’s Terra and Aqua satellites as 8-day composite values (Justice *et al*. 2002) and cattle densities. The use of temperature as a direct correlate of tsetse mortality rates, calculated in a number of different ways, has been successfully applied elsewhere (Rogers 1993, Hargrove 2001a,b, Hendrickx et al. 2001).

Cattle density was estimated using two variables: the monthly cattle density of a series of sentinel herds in the areas around the fly-rounds, and the general cattle density for the overall area.

The sentinel herds formed a small proportion of the total cattle population in the area, but are representative of the grazing patterns of all the cattle in the region. Longitudinal monitoring of these grazing patterns was carried out from February to November 2007 on four groups (one per site) each of 40 cattle (belonging to a total of 12 farmers). Monitoring involved taking geo-referenced GPS readings every approximately 100m, wherever the cattle were grazing, on three consecutive days per month, at monthly intervals. This monitoring was done by herdsmen who were trained to operate GPSs before the study began.

A monthly cattle density map of the sentinel animals was produced by applying a kernel Gaussian smoothing (Shawe-Taylor and Cristianini 2004) with a radius of 1.344 km around the monthly grazing points. This radius was derived from the tracking study; it is the average maximum distance (from the over-night kraal) covered by the cattle during grazing each day.

**References**

Hargrove, J.W. 2001a. The effect of climate on density-independent mortality in populations of male in Glossina m. morsitans in Zimbabwe and Tanzania. Bulletin of Entomological Research 91:79-86.

Hargrove, J.W. 2001b. Factors affecting density-independent survival of an island population of tsetse flies in Zimbabwe. Entomologia Experimentalis et Applicata 100:151-164.

Hendrickx ,G., A. Napala, J.H.W. Slingenbergh, R. De Deken, and D.J. Rogers. 2001. A contribution towards simplifying area-wide tsetse surveys using medium resolution meteorological satellite data. Bulletin of Entomological Research 91:333-346.

Justice, C.O., J.R.G. Townshend, E.F. Vermote, E. Masuoka, R.E. Wolfe, N. Saleous, D.P. Roy, and J.T. Morisette. 2002. An overview of MODIS Land data processing and product status. Remote Sensing of Environment 83:3-15.

Rogers, D.J., and S.E. Randolph. 1993. Distribution of tsetse and ticks in Africa: past, present and future. Parasitology Today 9:266-271.

Shawe-Taylor, J., and N. Cristianini. 2004. Kernel methods for pattern analysis. Cambridge University Press.
