# Supplementary material for: A Bayesian Geostatistical Moran Curve Model for Estimating Net Changes of Tsetse Populations in Zambia
Source: PLoS One. 2014 Apr 22;9(4):e96002. doi: 10.1371/journal.pone.0096002 (PMC3995969; doi:10.1371/journal.pone.0096002)
Supplement: Appendix S4 — Algorithm components. (DOC) [file pone.0096002.s004.doc]

**Appendix S4: Algorithm components.**

The Moran curve part of the algorithm was compiled in the R language (R Development Core Team 2012). The RAMPS sampling algorithm was taken from the R package “ramps” (Yan et al. 2007). The Markov Chain convergence statistics were taken from the R package “boa” (Smith 2007). Finally, the kriging was performed using the krige function of the R package “fields” (Furrer et al. 2012).

**References**

R Development Core Team. 2012. R: a language and environment for statistical computing. R Foundation for Statistical Computing, Vienna, Austria, ISBN 3-900051-07-0.

Furrer R., D. Nychka, and S. Sain. 2012. fields: Tools for spatial data. R package version 6.7. <http://CRAN.R-project.org/package=fields>.

Smith, B.J. 2007. boa: An R Package for MCMC Output Convergence Assessment and Posterior Inference. Journal of Statistical Software 11:1–37.

Yan, J., M.K. Cowles, S. Wang, and M. Armstrong. 2007. Parallelizing MCMC for Bayesian spatio-temporal geostatistical models. Statistics and Computing 17:323-335.
