# Supplementary material for: A Bayesian Geostatistical Moran Curve Model for Estimating Net Changes of Tsetse Populations in Zambia
Source: PLoS One. 2014 Apr 22;9(4):e96002. doi: 10.1371/journal.pone.0096002 (PMC3995969; doi:10.1371/journal.pone.0096002)
Supplement: Appendix S5 — Derivation of Equation 7 . (DOC) [file pone.0096002.s005.doc]

**Appendix S5: Derivation of Equation 7.**


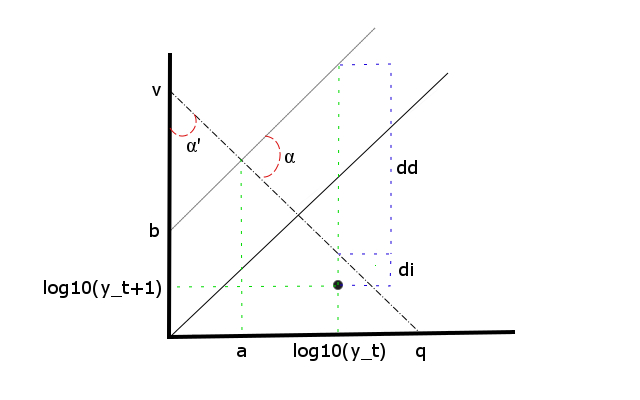


Fig E1. Moran Curve parameters: *y_t* and *y_t+1* are tsetse catches in successive months *t* and *t+1*and are plotted on logarithmic axes.The line from the origin of the axes is the ‘line of equality’ where successive monthly values are the same. The straight line with intercept *b*, parallel to the line of equality, defines the upper limit of population growth from one time interval to the next; line *vq* represents density dependent limitation of population growth; *a* is the point at which the density dependent mortality starts; *di* is one value of density independent mortality; *dd* is the density dependent mortality operating at the same time; and *α* is the angle between *vq* and the line of maximum population growth. The strength of density dependence depends on the value of *α*

Consider the line vq of the figure E1. This line has form:

log(yt+1)=v- tan α’ * log(yt) (1)

where α’ = 135° – α (since the line departing from b is at 45°)

where all the terms are known apart from v. The latter can be obtained from the known point a:

a+b=v – tan α’ * a (2)

v= a+b+ tan α’*a (3)

Since the line departing from b is parallel to the quadrant’s bisecting line:

dd=log(yt)+b- (log(yt+1)+di) (4)

(log(yt+1)+di) can be found solving the following equation:

(log(yt+1)+di)=v – tan α’*log(yt) (5)

Combining (4), (5) and (3) we obtain:

dd= log(yt)+b-a-b- tan α’ * a + tan α’*log(yt) (6)

removing b and grouping by tangα’

dd= log(yt)-a+ tan α’*(log(yt)-a) 7

That becomes:

dd = [log(y_t_)-a]*(1+ tan α’) 8
